# A Resolution-Free Parallel Algorithm for Image Edge Detection within the Framework of Enzymatic Numerical P Systems

**DOI:** 10.3390/molecules24071235

**Published:** 2019-03-29

**Authors:** Jianying Yuan, Dequan Guo, Gexiang Zhang, Prithwineel Paul, Ming Zhu, Qiang Yang

**Affiliations:** 1 The Postdoctoral Station at Xihua University Based on Collaboration Innovation Center of Sichuan Automotive Key Parts, Xihua University, Chengdu 610039, China; yuanjy@cuit.edu.cn (J.Y.); guodq@cuit.edu.cn (D.G.); 2 School of Control Engineering, Chengdu University of Information Technology, Chengdu 610225, China; zhuming@126.com; 3 School of Aeronautics and Astronautics, University of Electronic Science and Technology, Chengdu 610054, China; 4 Robotics Research Center, Xihua University, Chengdu 610039, China; qiangychd@126.com; 5 School of Electrical Engineering, Southwest Jiaotong University, Chengdu 610031, China; prithwineelpaul@gmail.com

**Keywords:** membrane computing, edge detection, enzymatic numerical P system, resolution free

## Abstract

Image edge detection is a fundamental problem in image processing and computer vision, particularly in the area of feature extraction. However, the time complexity increases squarely with the increase of image resolution in conventional serial computing mode. This results in being unbearably time consuming when dealing with a large amount of image data. In this paper, a novel resolution free parallel implementation algorithm for gradient based edge detection, namely EDENP, is proposed. The key point of our method is the introduction of an enzymatic numerical P system (ENPS) to design the parallel computing algorithm for image processing for the first time. The proposed algorithm is based on a cell-like P system with a nested membrane structure containing four membranes. The start and stop of the system is controlled by the variables in the skin membrane. The calculation of edge detection is performed in the inner three membranes in a parallel way. The performance and efficiency of this algorithm are evaluated on the CUDA platform. The main advantage of EDENP is that the time complexity of O(1) can be achieved regardless of image resolution theoretically.

## 1. Introduction

In recent decades, image processing technology has experienced dramatic growth and widespread applications. Nearly no area escapes impact in some way by digital image processing. Normally, digital image processing includes three main levels, i.e., low-level, mid-level and high-level processing [1]. As one of the most basic operators in low-level image processing, edge detection can preserve the important structural properties of an image while significantly reducing the amount of data. This excellent property makes it a basic tool for many high-level image processing algorithms and is extensively applied in target tracking [2], image compression [3], and object recognition [4]. An edge can be defined as points in a digital image at which the image brightness changes sharply or has discontinuities. This phenomenon may be caused by depth discontinuous, illumination changes, or intrinsic texture properties of objects. In various edge detection algorithms, the gradient based method is a type of classic edge detection approach with the merit of simple theory and good performance. However, as convolution calculation (i.e., a classic neighborhood computing in image processing) [5] is involved in this kind of algorithm, the time complexity increases squarely with the increase of image resolution. So it is difficult to deal with images with large resolution, such as remote sensing images, medical images, etc., in real time processing.

In order to achieve real-time calculation of high resolution images, many researchers have put much effort into this problem and several methods have been proposed. Generally, there are two main categories of resolutions. The first type of resolution concerns computational algorithms. In this kind of method, an elaboratively computational algorithm is usually designed to reduce the computational complexity. For the template matching problem, integral image [6] and dual-bound algorithm [7] are two classical approaches to speed up the computation. In [8], Fast LDA feature extraction is present, where steepest descent and conjugate direction methods are combined to optimize the step size in each iteration. In [9], common orthogonal basis extraction is proposed to extract a common basis of collection of matrices. The second category is based on hardware with parallel architecture, such as Graphics Processing Unit (GPU) [10,11,12] and Field Programmable Gate Array (FPGA) [13,14]. GPU uses hundreds of parallel processor cores executing tens of thousands of parallel threads to rapidly solve large problems having substantial inherent parallelism. However, with the shrinking volume of chips, semiconductor technology begins to reach its physical limits, which means the performance of conventional computing technique based on silicon chip integrated circuit microprocessors will be difficult to improve further [15]. Under this background, some scholars have turned their attention to non-traditional computing, such as quantum computing [16], DNA computing [17] and membrane computing (MC) [18]. MC is a new active branch of natural computing that simulates the function and structure of living cells and tissues, abstracting their biochemical reactions and material exchanges [19]. One of the most prominent features of MC is its capability of generating exponential growth space over a polynomial time, which makes it a promising method to resolve the conflict between the ever-increasing amount of data in the image processing field and the backward computing power of conventional computer [20]. In recent years, image edge detection and image segmentation [21,22,23,24], image smoothing [25], obtaining homology groups of 2D images [26,27], counting cells [28], Enzymatic numerical P systems image thinning [29] and corner detection [30] in MC framework have been vividly studied. In the previous literature about MC and image processing, much work is based on tissue-like P systems. However, when designing a parallel implementation program of an existing image processing algorithm, it is difficult to realize the mathematical formula in “tissue-like P systems language”. The reasons for this are as follows. First, the data type of an image is an integer between 0 and 255. When design image processing algorithm uses tissue-like P systems, the image data should be coded to symbolic variables and those symbolic variables need to be decoded to integer for display as the algorithm finished. Second, most image processing algorithms are composed of several steps in determined logical order, which means variables in the membrane system need to be calculated in a deterministic way, rather than in a random manner. Since the rules in tissue-like P systems are implemented randomly, it is difficult to control the execution orders of different rules.

In order to overcome the above shortcomings when tissue-like P systems are combined to image processing, we make the first attempt to introduce enzymatic numerical P system (ENPS) to image processing. Concretely, a parallel algorithm for gradient based edge detection algorithm is designed and tested in the framework of ENPS. Besides the features described in [25], ENPS has another two good properties which make it particularly appropriate for image processing. One is that numerical variables and numerical expressions can be used directly in ENPS. Thus, image data can be directly operated without the additional encoding and decoding process. Another important characteristic is that enzymatic variables can control the execution orders of multiple rules in ENPS, i.e., the algorithms with complex logical steps can be designed easily.

The main contribution of this paper is that a parallel algorithm for image edge detection in the framework of ENPS, namely EDENP, is designed. The significant advantage of EDENP is that it can achieve the time complexity of O(1) theoretically, no matter how large the image resolution is. Moreover, the performance is equivalent to the performance run on the serial computing platform. This is very important for real projects, because most of the classical image processing algorithms have been widely proven to be effective in practical engineering, so the designed parallel implementation algorithm can be directly applied to the real image processing project without the need to perform large-scale testing. To the best of our knowledge, it is the first time to bridge problems from image processing with ENPS.

The rest of this paper is structured as follows. Section 2 introduces the definition, characteristics and applications of MC and ENPS. The problem statement is elaborated in Section 3. Section 4 discusses the EDENP algorithm in detail. The experiments and results are presented in Section 5. Conclusions are drawn in Section 6.

## 2. MC and ENPS

MC is a young biocomputing model proposed by Gh.Păun in 2000 [19]. The computational devices in MC are called P systems. Generally, a P system includes three ingredients: (i) the membrane structure; (ii) multisets of objects; (iii) rules of a bio-chemical inspiration. The multisets of objects are placed in the membrane, and evolved according to given rules which are usually applied in a synchronous non-deterministic maximally parallel manner. Since being proposed, MC has received great attention from scientists in many fields [31,32,33]. In the past 20 years, both the theory [32,34,35,36,37] and application [31,38,39,40,41] of MC have been greatly developed, and many different classes of P systems have been investigated. According to the way in which membranes are structured, there are three major types of P systems, i.e., cell-like [19], tissue-like [42] and spiking neural P systems [43,44]. Enzymatic numerical P system comes from numerical P system (NPS). NPS is a new special research branch of cell-like P systems, proposed by the founder of MC, Gh.Păun in 2006 [45]. In NPS, multisets of objects associated to membranes are sets of numerical variables, and the evolutionary rules are composed of a production function and a repartition protocol [46,47,48]. The most common widely application area of NPS is robot controller design [49,50,51,52]. Although NPS can deal with numerical variables, it can only execute one production function per membrane at a time. When there are multiple production functions per membrane, one is selected randomly. This limits its application in some situations where the rules should be executed deterministically. In order to solve this problem and expand the application of NPS, ENPS is put forward [24]. It is extended from NPS by introducing enzyme-like variables which can make rules run deterministically [53]. The standard form of ENPS is defined as follows:

$$\Pi =\left(m,H,\mu ,\left(Var_{1},E_{1},{\mathrm{Pr}}_{1},Var_{1}\left(0\right)\right),\ldots ,\left(Var_{m},E_{m},{\mathrm{Pr}}_{m},Var_{m}\left(0\right)\right)\right).$$

where:

1. *m* is the number of membranes used.
2. *H* is an alphabet that contains *m* symbols, and H={1,2,…,m}.
3. μ is the membrane structure.
4. $Var_{i}$ is the set of variables from membrane *i* and $Var_{i}\left(0\right)$ are the initial values for these variables.
5. ${\mathrm{Pr}}_{i}$ is the set of rules in membrane *i*, composed of a production function and a repartition protocol. A typical rule is as follows.
  $${F_{l,i}\left(y_{1,i},\ldots ,y_{k_{l},i}\right)|}_{e_{j,i}}\to c_{l,1}|v_{1}+c_{l,2}\left|v_{2}+\ldots +c_{l,n_{i}}\right|v_{n_{i}},$$
  where $e_{j,i}$ is a variable from $Var_{i}$ different from $y_{1,i},\ldots ,y_{k_{l},i}$ and $v_{1},v_{2},\ldots ,v_{n_{i}}$. The rule can be executed at a time *t* only if $e_{j,i}>\min\left\{y_{1,i}\left(t\right),y_{2,i}\left(t\right),\ldots y_{k_{l},i}\left(t\right)\right\}$. From the definition of ENPS, it is clear that with enzymes-like variables, the system can control multiple production functions to run in parallel in the same membrane deterministically [54]. Hence, it can overcome the disadvantages of traditional NPS that only run one rule nondeterministically at a time in a membrane. The ENPS with deterministic, parallel execution model has already been proved to be Turing universal [55,56]. In [57], it is shown that any ENPS working in all-parallel mode or one parallel model can be simulated by an equivalent one-membrane ENPS working in the same mode. Since the proposal of ENPS, this model has been successfully applied in a wide range of domains, such as robot control [58], big data field [59], and sequential minimal optimization [60] fields. In this paper, ENPS is used to solve the problem of gradient based image edge detection.

## 3. Problem Statement

Edges generally occur in areas where the brightness of the image changes dramatically. These changes can be described by image gradients. Usually, a pair of convolution masks are used to estimate the gradients in the *x* and *y* directions, respectively, as shown in Equations (1)–(3), where ($Sobel_{x}$, $Sobel_{y}$), ($Prew_{x}$, $Prew_{y}$), ($Rob_{x}$, $Rob_{y}$) are three classic pairs of convolution masks. In this paper, we take Sobel operator as an example of gradient based edge detection (GBED). When the masks are sliding over the image, a square of pixels are operated. Then both directional gradients and absolute gradient magnitudes of image are computed, as shown in Equations (4) and (5), where *I* is the image, ($g_{x}$, $g_{y}$) are gradients in *x* and *y* direction respectively, $g_{i,j}$ is the absolute gradient magnitude of a pixel with coordinate (i,j), 2≤i,j≤n−1 for image with resolution of n×n.

(1) $$Sobel_{x}=\left[\begin{array}{ccc} -1 & 0 & 1\\ -2 & 0 & 2\\ -1 & 0 & 1 \end{array}\right];\;Sobel_{y}=\left[\begin{array}{ccc} 1 & 2 & 1\\ 0 & 0 & 0\\ -1 & -2 & -1 \end{array}\right]$$

(2) $$Prew_{x}=\left[\begin{array}{ccc} -1 & 0 & 1\\ -1 & 0 & 1\\ -1 & 0 & 1 \end{array}\right];\;Prew_{y}=\left[\begin{array}{ccc} 1 & 1 & 1\\ 0 & 0 & 0\\ -1 & -1 & -1 \end{array}\right]$$

(3) $$Rob_{x}=\left[\begin{array}{cc} -1 & 0\\ 0 & 1 \end{array}\right];\;Rob_{y}=\left[\begin{array}{cc} 0 & -1\\ 1 & 0 \end{array}\right]$$

(4) $$g_{x}=Sobel_{x}*I;\;g_{y}=Sobel_{y}*I;$$

(5) $$g_{i,j}=\sqrt{{g_{x_{i,j}}}^{2}+{g_{y_{i,j}}}^{2}}$$

When the gradient magnitude $g_{i,j}$ is computed, the difference between it and a predefined threshold θ is used to judge whether this pixel is an edge pixel or not, as presented in Equation (6), where $d_{i,j}$ is the difference. More concretely, if $d_{i,j}$ is greater than or equal to 0, then the pixel is assumed as an edge point, otherwise, it is a background point, as shown in Equation (7). It is worth noting that in real application, before thresholding, the gradient image should be filtered by “non-maximum suppression” for getting more real edges. In this paper, in order to simplify the algorithm, this step is ignored.

(6) $$d_{i,j}=g_{i,j}-\theta$$

(7) $$edg_{i,j}=\{\begin{array}{cc} 1 & \mathrm{if}\;(d_{i,j}\geq 0)\\ 0 & \mathrm{if}\;(d_{i,j}<0) \end{array}$$

The program pseudo code of GBED run on conventional serial computer is illustrated in Algorithm 1, where the initial value of $edg_{i,j}$ is set to 0. From Algorithm 1, it can be deduced that the computational complexity is $O(n^{2})$ because two loops are involved. When *n* becomes large, the calculations are very time-consuming under the serial computing platform.
**Algorithm 1:** The pseudo code of GBED**Input:** I(n∗n)**Output:** edg(n∗n) 1:**for**i=2:n−2**do** 2: **for** j=2:n−1 **do** 3:  Computing $g_{x_{i,j}}$ 4:  Computing $g_{y_{i,j}}$ 5:  Computing $g_{i,j}$ 6:  Computing $d_{i,j}$ 7:  Computing $edg_{i,j}$ 8: **end for** 9:**end for**

In order to reduce the calculation time complexity, we attempt to introduce an enzymatic numerical P system to design a high parallel computing algorithm for edge detection. The details of how to design the algorithm will be given in the next section.

## 4. The EDENP Algorithm

This section starts with the mathematical model of EDENP followed by the detailed description of EDENP. The execution process and resources needed are discussed lastly.

### 4.1. Mathematical Model of EDENP

**Figure 1:**
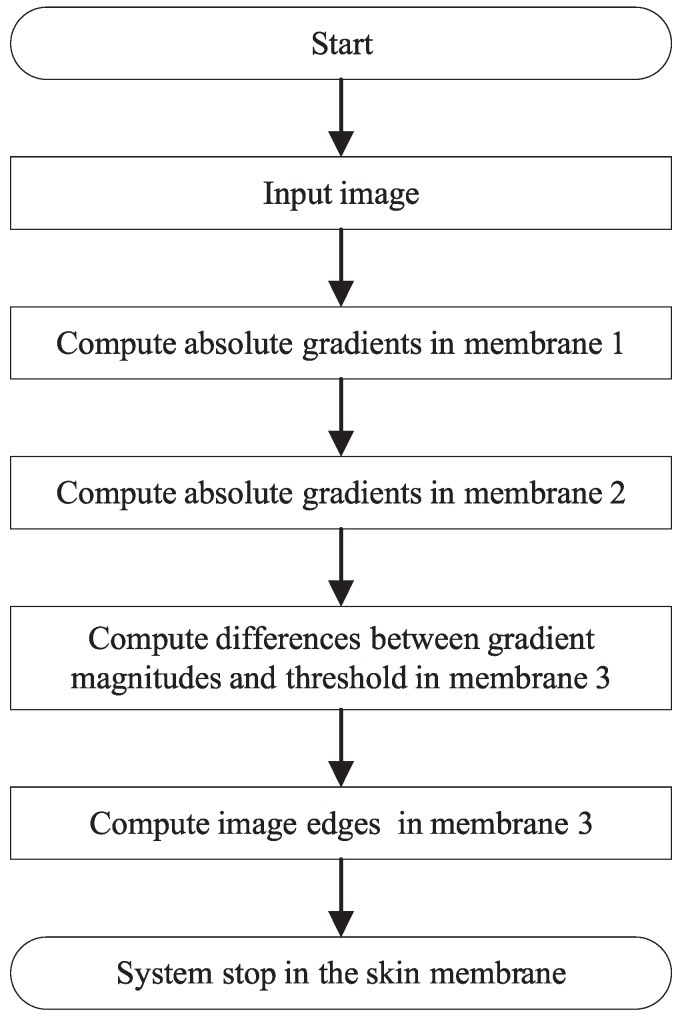
The flowchart of EDENP.

**Figure 2:**
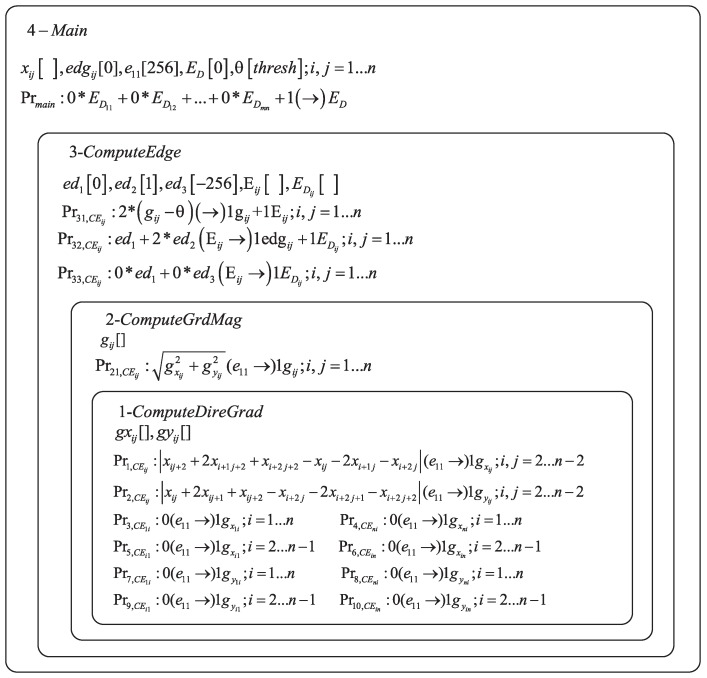
The structure of EDENP.

From Section 3, we know that the GBED algorithm contains four steps for a certain pixel in an image. In EDENP, the four steps will be executed in a cell-like P system under the control of enzyme variables, as illustrated in Figure 1. The initialization of variables, start and stop of the system will be controlled in the skin membrane. The directional gradients estimation will be completed in membrane 1. The absolute gradient magnitude estimation will take place in membrane 2. Membrane 3 is responsible for computing the image edge. The corresponding membrane structure is illustrated in Figure 2.

The mathematical expression of EDENP is as follows, and

$$\Pi =\left(m,H,\mu ,\left(Var_{1},E_{1},{\mathrm{Pr}}_{1},Var_{1}\left(0\right)\right),\ldots ,\left(Var_{4},E_{4},{\mathrm{Pr}}_{4},Var_{4}\left(0\right)\right)\right),$$

where

1. m=4.
2. H={1,2,3,4}.
3. $u=[[[[\;\;{]}_{1}{]}_{2}{]}_{3}{]}_{4}$.
4. $Var_{1}=\{g_{x_{i,j}},g_{y_{i,j}}\}$, $Var_{2}=g_{i,j}$, $Var_{3}=\left\{ed_{1},ed_{2},ed_{3},E_{i,j},E_{D_{i,j}}\right\}$, $Var_{4}=\left\{x_{i,j},edg_{i,j},\theta ,e_{1,1},E_{D}\right\}$.
  $x_{i,j}\left(1\leq i,j\leq n\right)$, are the gray value of pixel with coordinate of (i,j) on the source image plane.
  $edg_{i,j}\left(1\leq i,j\leq n\right)$, are the corresponding edge points of the source image with initial value 0.
  θ[threshold], is a numerical variable which is used as the threshold value for edge detection, and the value of threshold should be predefined.
  $g_{x_{i,j}}\left(1\leq i,j\leq n\right)$, are the horizontal derivative approximations at each pixel.
  $g_{y_{i,j}}\left(1\leq i,j\leq n\right)$, are the vertical derivative approximations at each pixel.
  $g_{i,j}\left(1\leq i,j\leq n\right)$, are the gradient magnitude approximations at each pixel.
  $ed_{1}\left[0\right]$, is a numerical variable with initial value 0, which is used as the background value of the edge image.
  $ed_{2}\left[1\right]$, is a numerical variable with initial value 1, which is used as the edge point value of the edge image.
  $ed_{3}\left[-256\right]$, is a numerical variable with initial value −256, which is used as a intermediate variable.
5. $E_{k}$ is a set of enzyme variables from membrane *k*, i.e., $E_{1}=\left[\;\;\right]$,$E_{2}=\left[\;\;\right]$,$E_{3}=\left\{E_{i,j},E_{D_{i,j}}\right\}$,$E_{4}=\left\{e_{1,1},E_{D}\right\}$.
6. ${\mathrm{Pr}}_{k}$ is the set of programs (rules) in membrane *k*, composed of a production function and a repartition protocol.
  ${\mathrm{Pr}}_{1,CE_{i,j}}:\;\left(\left|x_{i,j+2}+2x_{i+1,j+2}+x_{i+2,j+2}-x_{i,j}-2x_{i+1,j}-x_{i+2,j}\right|\right){|}_{e_{1,1}}\to 1|g_{x_{i,j}},(2\leq i,j\leq n-2),$
  ${\mathrm{Pr}}_{2,CE_{i,j}}:\;\left(\left|x_{i,j}+2x_{i,j+1}+x_{i,j+2}-x_{i+2,j}-2x_{i+2,j+1}-x_{i+2,j+2}\right|\right){|}_{e_{1,1}}\to 1|g_{y_{i,j}},(2\leq i,j\leq n-2),$
  ${\mathrm{Pr}}_{3,CE_{1,i}}:0{|}_{e_{1,1}}\to |g_{x_{1,i}};\left(1\leq i\leq n\right)$,
  ${\mathrm{Pr}}_{4,CE_{n,i}}:0{|}_{e_{1,1}}\to |g_{x_{n,i}};\left(1\leq i\leq n\right)$,
  ${\mathrm{Pr}}_{5,CE_{i,1}}:0{|}_{e_{1,1}}\to |g_{x_{i,1}};\left(2\leq i\leq n-1\right)$,
  ${\mathrm{Pr}}_{6,CE_{i,n}}:0{|}_{e_{1,1}}\to |g_{x_{i,n}};\left(2\leq i\leq n-1\right)$,
  ${\mathrm{Pr}}_{7,CE_{1,i}}:0{|}_{e_{1,1}}\to |g_{y_{1,i}};\left(1\leq i\leq n\right)$,
  ${\mathrm{Pr}}_{8,CE_{n,i}}:0{|}_{e_{1,1}}\to |g_{y_{n,i}};\left(1\leq i\leq n\right)$,
  ${\mathrm{Pr}}_{9,CE_{i,1}}:0{|}_{e_{1,1}}\to |g_{y_{i,1}};\left(2\leq i\leq n-1\right)$,
  ${\mathrm{Pr}}_{10,CE_{i,n}}:0{|}_{e_{1,1}}\to |g_{y_{i,n}};\left(2\leq i\leq n-1\right)$.
  Those rules are used to execute Formula (1). The enzyme in ${\mathrm{Pr}}_{1,CE_{i,j}}∼{\mathrm{Pr}}_{10,CE_{i,j}}$ must exist in enough amount so that the rules can be activated. Specifically, if the value of the enzyme $e_{1,1}$ is greater than variable $x_{i,j}\left(1\leq i,j\leq n\right)$, then rules ${\mathrm{Pr}}_{1,CE_{i,j}}$$∼{\mathrm{Pr}}_{10,CE_{i,j}}$ are effective. Since variable $x_{i,j}$ is the gray value of image, the maximum value is 255. So, the initial value of $e_{1,1}$ is set to 256, such that the condition modeled by rule ${\mathrm{Pr}}_{1,CE_{i,j}}∼{\mathrm{Pr}}_{10,CE_{i,j}}$ are satisfied. It is important to note that the number of rules are n×n, and all the rules are executed in parallel.
7. ${\mathrm{Pr}}_{21,CE_{i,j}}:\left(\sqrt{g_{x_{i,j}}^{2}+g_{y_{i,j}}^{2}}\right)\left|e_{1,1}\to 1\right|g_{i,j};1\leq i,j\leq n$
  ${\mathrm{Pr}}_{21,CE_{i,j}}$ are the rules which are executed by Formula (5). Hence, after executing ${\mathrm{Pr}}_{1,CE_{i,j}}$$∼{\mathrm{Pr}}_{10,CE_{i,j}}$, the value of the variables $g_{x_{i,j}}$, $g_{y_{i,j}}$ are obtained. The maximum value of $g_{x_{i,j}}$ and $g_{y_{i,j}}$ is 255, and the enzyme $e_{1,1}$ is 256. So the condition of execution for rules ${\mathrm{Pr}}_{21,CE_{i,j}}$ is satisfied. Hence, all n×n rules are executed concurrently.
8. ${\mathrm{Pr}}_{31,CE_{i,j}}:\left(2*\left(g_{i,j}-\theta \right)\right)\left|\to 1\right|g_{i,j}+1|E_{i,j};1\leq i,j\leq n$
  ${\mathrm{Pr}}_{31,CE_{i,j}}$ are the rules which compute $d_{i,j}$ in Formula (6). After executing ${\mathrm{Pr}}_{31,CE_{i,j}}$, the value of $d_{i,j}$ are obtained, which is equal to variables $g_{i,j}$ and $E_{i,j}$ in rule ${\mathrm{Pr}}_{31,CE_{i,j}}$.
9. ${\mathrm{Pr}}_{32,CE_{i,j}}:\left(ed_{1}+2*ed_{2}\right){|}_{E_{i,j}}\to 1\left|\mathrm{ed}g_{i,j}+1\right|E_{D_{i,j}}$; ${\mathrm{Pr}}_{33,CE_{i,j}}:\left(0*ed_{1}+0*ed_{3}\right){|}_{E_{i,j}}\to 1\left|\mathrm{ed}g_{i,j}+1\right|E_{D_{i,j}};\;1\leq i,j\leq n.$
  ${\mathrm{Pr}}_{32,CE_{i,j}}$ and ${\mathrm{Pr}}_{33,CE_{i,j}}$ are rules for computing edge value as Formula (7). If $E_{i,j}$ is greater than or equal to 0, then ${\mathrm{Pr}}_{32,CE_{i,j}}$ and ${\mathrm{Pr}}_{33,CE_{i,j}}$ are executed. Because $ed_{1}$ is 0, and $ed_{3}$ is -256, so $E_{i,j}\geq \min\left(ed_{1},ed_{2}\right)$ and $E_{i,j}\geq \min\left(ed_{1},ed_{3}\right)$. The execution condition of ${\mathrm{Pr}}_{32,CE_{i,j}}$ and ${\mathrm{Pr}}_{33,CE_{i,j}}$ is satisfied. If $d_{i,j}<0$, only ${\mathrm{Pr}}_{33,CE_{i,j}}$ will be executed. Because $E_{i,j}\geq \min\left(ed_{1},ed_{3}\right)$ and $E_{i,j}<\min\left(ed_{1},ed_{2}\right)$, only the execution condition of ${\mathrm{Pr}}_{33,CE_{i,j}}$ can be satisfied. After executing ${\mathrm{Pr}}_{32,CE_{i,j}}$ and ${\mathrm{Pr}}_{33,CE_{i,j}}$, variables $edg_{i,j}$ will be set to 1 if $d_{i,j}\geq 0$ and every variable $E_{D_{i,j}}$ will be assigned.
10. ${\mathrm{Pr}}_{main}:\left(0*E_{D_{1,1}}+0*E_{D_{1,2}}+\ldots +0*E_{D_{m,n}}+1\right)\left|\to 1\right|E_{D}$
  ${\mathrm{Pr}}_{main}$ is a rule contained in membrane 4, which controls the stop condition of the P system. For pixel (i,j), if all the enzyme variables $E_{D_{i,j}}$ are assigned, the condition for ${\mathrm{Pr}}_{main}$ is meet. Enzyme variable $E_{D}$ is set to 1 by rule ${\mathrm{Pr}}_{main}$, and the system stops running.

### 4.2. The Structure and Execution Processes of EDENP

As shown in Figure 2, the structure of EDENP includes four membranes. The system begins to start when the input variables $x_{i,j}$ representing the gray value of source image at location (i,j) appear in the skin membrane. The whole process includes five steps.

Step 1: Horizontal and vertical derivative approximations of every pixel are computed in membrane 1 by using rules of ${\mathrm{Pr}}_{1,CE_{i,j}}∼{\mathrm{Pr}}_{10,CE_{i,j}}$ in a parallel manner. When the directional gradients are computed, membrane 2 will be activated.

Step 2: The gradient magnitude of all the pixels are obtained at the same time with rules of ${\mathrm{Pr}}_{21,CE_{i,j}}$ in membrane 2.

Step 3: The comparisons between the gradient magnitudes of all pixels and the predefined threshold are executed by rules of ${\mathrm{Pr}}_{31,CE_{i,j}}$ in membrane 3.

Step 4: The edge pixels are detected and marked with 1, while the background pixels are marked with 0 by rules of ${\mathrm{Pr}}_{32,CE_{i,j}}$ and ${\mathrm{Pr}}_{33,CE_{i,j}}$ in membrane 3.

Step 5: The system stop condition is satisfied and the system stops working by rules of ${\mathrm{Pr}}_{main}$ in membrane 4.

So as described above, only five steps are needed in the proposed algorithm for images with arbitrary resolution. Since we do not change the mathematical model of Sobel based edge detection, the detection result by our proposed method is the same as if run on a serial computing platform.

### 4.3. Complexity and Resources Analysis

Taking into account that the size of the input data is n×n, and the image is a gray image. The amount of resources needed is illustrated in Table 1. From Table 1, we can see that there are $\left(7n^{2}+6\right)$ variables, including $\left(2n^{2}+2\right)$ enzymatic variables and $\left(5n^{2}+4\right)$ numerical variables. $\left(6n^{2}+4\right)$ rules are involved in this system. The total storage space is 1 cell with $\left(13n^{2}+10\right)$ molecules. So the space complexity is $O(n^{2})$ theoretically. The time complexity is O(1) because the number of execution steps is 5, which implies the computational efficiency is constant for images under arbitrary resolutions.

From the above analysis, we can see that the core of the proposed algorithm is to use space to replace time to obtain high-performance parallel computing, which is exactly the prominent characteristic of MC. Since molecules are used as storage units in a real biological computer, huge storage space can be utilised when this algorithm is implemented on it. So we think the proposed parallel algorithm is effective for images with high resolutions, at least at a theoretical level.

## 5. Experiments and Results

In this section, both the performance and efficiency of our proposed EDENP algorithm are evaluated. Since there is no hardware implementation of MC systems at present, the only way to test the behaviors of the designed P systems is to simulate them in conventional computers. In this paper, a parallel computing architecture, Compute Unified Device Architecture (CUDA), is used as the simulating platform, as it has been reported in literature [24,61]. The parameters of the platform on which our experiments are carried out are illustrated in Table 2. The threshold θ for all the experiments is set to 0.2.

### 5.1. Performance Evaluation

Two case studies are considered to evaluate the performance of the proposed method for different types of images. Since the proposed algorithm is in the framework of MC, edge detection methods based on tissue-like *P* systems [21,24] are chosen as comparison methods. Algorithms in the literature [21,24] are sketched and implemented on a CPU platform using the MATLAB program.

#### 5.1.1. Qualitative Evaluation

**Figure 3:**
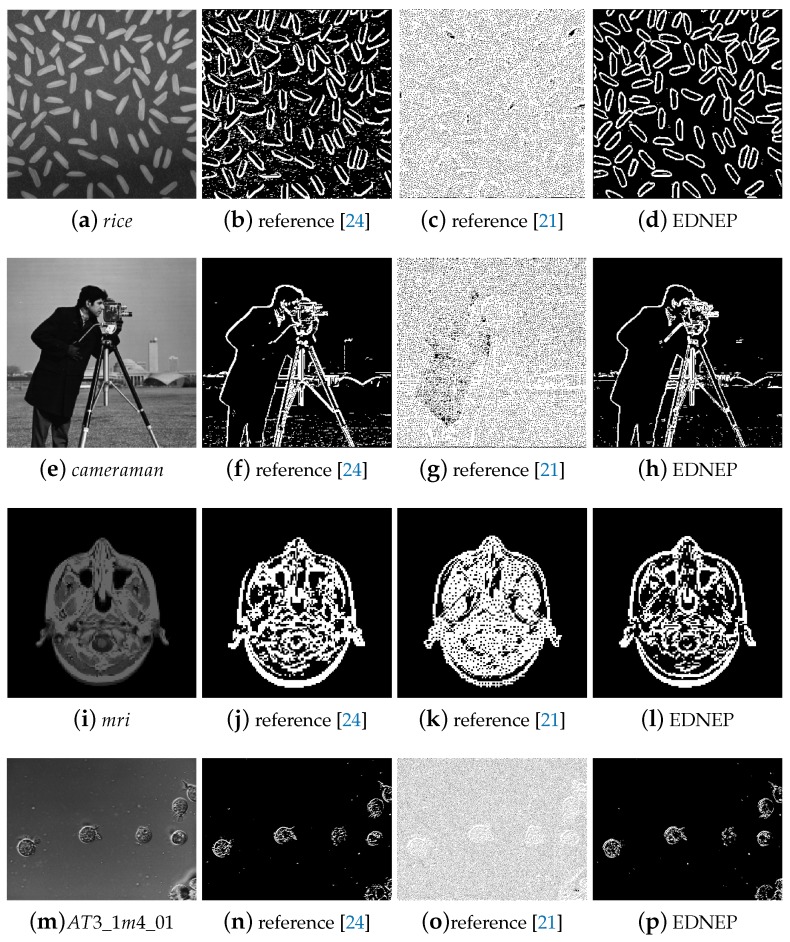
Edge detection results of images with rich texture (the first column: the source gray images; the second to the last column: results by using methods in [21,24] and EDENP respectively).

Case study 1 is considered to evaluate the performance of the three algorithms for images with rich textures. Four images named *rice*, *cameraman*, *mri*, and *AT3_lm4_01* randomly collected from the MATLAB Image Tool Box are used as testing samples in this experiment, as shown in Figure 3a,e,i,m. Figure 3b–d,f–h,j–l,n–p show the detailed qualitative edge detection results of the three algorithms for the four images. It can be clearly observed from Figure 3b,f,j,n, that the contours of the objects can be detected, but meanwhile the noise in the background is also detected, which will make the following image processing, such as object recognition, more difficult to deal with. The results by reference [21] are shown in Figure 3c,g,k,o. It can be seen that there are too many small edges, and the main outlines of the targets can hardly be found even by human eyes. The results of EDENP are illustrated in Figure 3d,h,l,p, from which we can see that not only the main contours of objects can be detected successfully, but also the noise is well suppressed.

**Figure 4:**
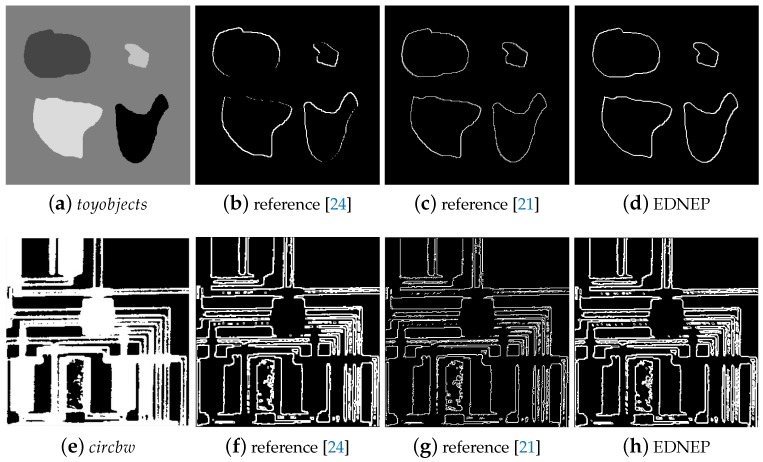

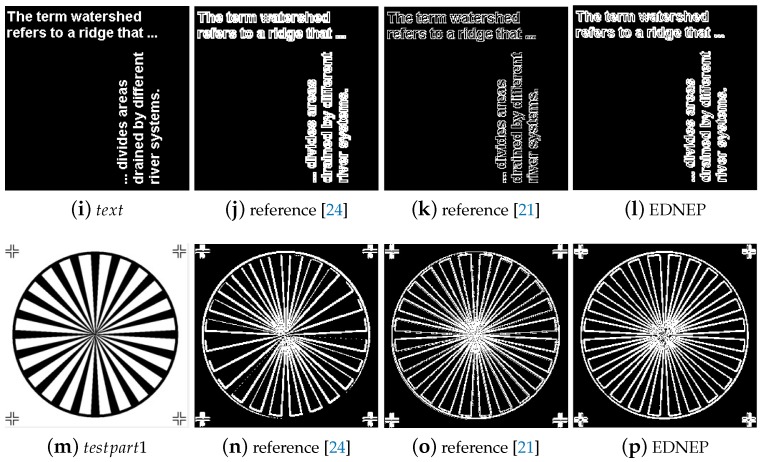
Edge detection results of images with less texture (the first column: the source gray images; the second to the last column: results by using methods in [21,24] and EDENP, respectively).

**Figure 5:**
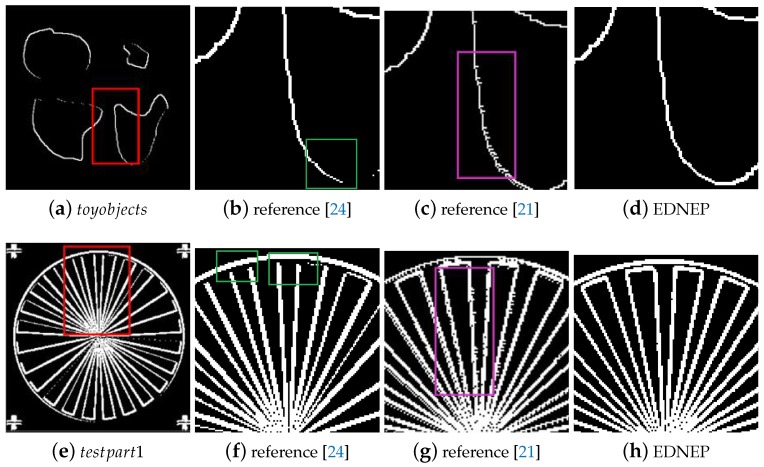
Edge detection results of toyobjects and testpart1 (the first column: the edge image; the second to the last columns: the local edge image enlarged by using methods in [21,24] and EDENP respectively).

Case study 2 is used to test the performance of the three methods for images with less texture, in which images named *toyobjects*, *circbw*, *text*, *testpart1* randomly selected from MATLAB Image Tool Box are used as testing image samples. In image *toyobjects*, each object has a constant gray value, while the other three images are binary images. Like in Case 1, the detected edge results by the three approaches are shown in Figure 4. Figure 4b,f,j,n clearly show that there are many discontinuous edges when using algorithm in reference [24], while the other two methods can detect the edges completely. When comparing the thickness of the edges, it is obvious to see that the method in reference [21] can achieve the thinnest edges, then the EDENP method, and the edges detected by [24] is the thickest. Although the method in [21] can obtain the finest edges, those edges often have burrs, as shown in Figure 5. Figure 5a,e are the whole edge image of toyobjects and circbw. Figure 5b–d,f–h are the local enlargement of areas in pink rectangles in Figure 5a,e. Areas marked in green in Figure 5b,f are some examples of discontinuous edges by [24]. When comparing Figure 5c,g with Figure 5d,h, it is clear that edges by EDENP are much smoother than by algorithm [21].

#### 5.1.2. Quantitative Evaluation

The confidence degree of the edge image is one of the most used indexes for evaluating the authenticity of the edge pixels. In general, the greater the edge confidence degree is, the more reliable the edges are. In this paper, we use this index to evaluate the performance of the edge detection algorithm quantitatively, whose mathematical definition is presented in reference [62].

Table 3 provides the comparison results of the three methods in terms of edge confidence degree. It can be seen from Table 3 that the EDENP method has the highest edge confidence degree for images with both high and low texture, which means edges detected by EDENP have less false edges.

Through the above quantitative and qualitative results, it can be deduced that the method in reference [21] is nearly invalid for grayscale images with rich texture. For images with less textures, this method can get the fine edges of the objects. However, the edges are not smooth in some cases because of the false burr edge points. The approach in [24] cannot get the whole contours of the objects due to the discontinous edges detected for images with both rich and less rich textures. The EDENP algorithm has the highest performance and can obtain clear, continuous, and authentic edges of images with both rich and less rich textures.

In this paper, only edge detection methods in the framework of MC are chosen for a comparison. From the above experimental results, we can see that the proposed algorithm has better performance compared with the existing tissue-like based edge detection methods. The fundamental reason for this is that with the help of “enzyme variables” in ENPS, the rules can be controlled flexibly, thus the existing Sobel edge detection algorithm can be programmed in “membrane computing language” easily.

### 5.2. Efficiency Evaluation

To better describe the computation efficiency of EDENP, a speedup ratio is defined as the elapsed time of algorithm on CPU platform divided by running time on GPU platform. The running times of images with different resolutions under GPU and CPU platform and corresponding speedup ratios for one image (*camera*) are illustrated in Table 4. From Table 4, we can see, although the computation times of EDENP are independent of resolutions theoretically, it takes different times to execute the EDENP algorithm for the same image at different resolutions. The reason for this is that the programs do not run on real bio-computers. Table 5 gives the speedup ratios results of the other seven images. It can be found that the lowest speedup is 53, and the maximum speedup can reach up to 262. It is obvious that the computing power of the proposed algorithm is much superior compared with the traditional algorithm implemented on CPU platform.

## 6. Conclusions

Membrane computing is a new branch of natural computing, and its amazing storage space and high parallel computing characteristics are very suitable for big data processing. Among various membrane systems, the ENPS can directly deal with numeric variables, and the enzyme variables can flexibly control the execution orders of different rules. In this paper, we attempt to apply ENPS to image processing, and take Sobel edge detection as an example. Compared with the previous works which are based on tissue-like P systems, the advantage of the proposed method is that it does not need to encode and decode the image data, and it is easy to write the program for algorithms with complex execution orders in “membrane computing language”. The limitation of the proposed algorithm mainly has two aspects. One is that the execution of the algorithm is based on real biological computers. However, there are no universal biological computers at present, so it is difficult to evaluate the real computing efficiency of the proposed algorithm. The other shortage is that the space complexity is $O(n^{2})$, which means large storage space is needed for the proposed algorithm. In future research, we will simulate the algorithm on FPGA hardware and try to combine the ENPS with other, more complex image processing algorithms.

## Figures and Tables

**Table 1:** Complexity and resources needed for EDENP.

Term	Necessary Resources
Initial number of cells	1
Number of enzymatic variables	$2n^{2}+2$
Number of numerical variables	$5n^{2}+4$
Number of rules	$6n^{2}+4$
Execution steps	5

**Table 2:** Parameters the computer used.

Term	Parameters
CPU model	Intel(R) Core(TM) i7-7700HQ
cache memory	8 MB, 16-Way, 64 byte lines
main memory	16 GB (2* DDR4 2400MHz)
hard disc	SSD, SK hynix SC308 SATA 128GB, 600 Mbps; MQ01ABD100, 1TB
GPU model	Nvidia GeForce GTX 1050 Ti (4 GB)
execution steps	5

**Table 3:** The edge confidence degree.

	Reference [24]	Reference [21]	EDENP
*rice*	0.75	0.56	**0.84**
*cameraman*	0.66	0.32	**0.74**
*mri*	0.63	0.56	**0.68**
*AT3_lm4_01*	0.44	0.12	**0.5**
*toyobjects*	0.85	0.76	**0.86**
*circbw*	0.94	0.93	**0.95**
*text*	0.93	0.90	**0.94**
*testpart1*	0.81	0.79	**0.86**

**Table 4:** Elapsed time of images with different resolution (cameraman).

Image Resolution	$256^{2}$	$384^{2}$	$512^{2}$	$768^{2}$	$1024^{2}$	$2048^{2}$	Platform
Elapsed time(ms)	0.014	0.03	0.05	0.12	0.23	0.86	GPU
Elapsed time(ms)	3.5	9.1	4.4	9.6	41.9	72.8	CPU
Speedup ratio	250	303	88	80	182	130	

**Table 5:** The speedup ratio of seven images.

Image Resolution	$256^{2}$	$384^{2}$	$512^{2}$	$768^{2}$	$1024^{2}$	$2048^{2}$	
*rice*	79	121	79	101	136	82	
*mri*	60	80	77	62	73	66	
*AT3_lm4_01*	80	90	102	172	71	75	
*toyobjects*	187	162	163	81	182	62	
*circbw*	193	213	262	210	176	66	
*text*	167	180	194	118	57	65	
*testpart1*	53	76	100	161	87	64	

## Supplementary Materials

Supplementary Materials are available online.
